# Protocol for label-free quantitative lysine lactylproteome profiling

**DOI:** 10.1016/j.xpro.2025.103726

**Published:** 2025-04-03

**Authors:** Yu Ran, Qiqing Yang, Lianqi Zhou, Heyu Li, Bing Yang, Long Zhang

**Affiliations:** 1 Department of Radiation Oncology and the State Key Laboratory of Transvascular Implantation Devices, the Second Affiliated Hospital, School of Medicine, Zhejiang University, Hangzhou, China; 2 The MOE Basic Research and Innovation Center for the Targeted Therapeutics of Solid Tumors, The First Affiliated Hospital, Jiangxi Medical College Nanchang University, Nanchang, China; 3 Frontiers Medical Center, Tianfu Jincheng Laboratory, Chengdu, China

**Keywords:** Proteomics, Protein expression and purification, Mass spectrometry

## Abstract

L-lactate has been recognized as an essential molecule for signaling and metabolic balance. Lactylation, a post-translational modification (PTM) derived from L-lactate, is commonly observed on various proteins and plays essential roles in cellular processes. Here, we present a protocol to globally profile the lactylation proteome and perform label-free quantification. We provide steps for cell preparation, protein extraction, digestion, peptide desalting, and lactylpeptide enrichment. Additionally, we outline the parameters for liquid chromatography-tandem mass spectrometry (LC-MS/MS) analysis.

For complete details on the use and execution of this protocol, please refer to Li et al.^1^

## Highlights

• Instructions for the preparation of cell culture, transfection, and lactate treatment
• Steps for enriching lactylpeptide based on antibody-conjugated agarose beads
• Guidance on LC-MS setup and MaxQuant parameters for label-free quantification analysis

## Abstract (editor highlights)

Publisher’s note: Undertaking any experimental protocol requires adherence to local institutional guidelines for laboratory safety and ethics.

## Graphical abstract

**Figure:**
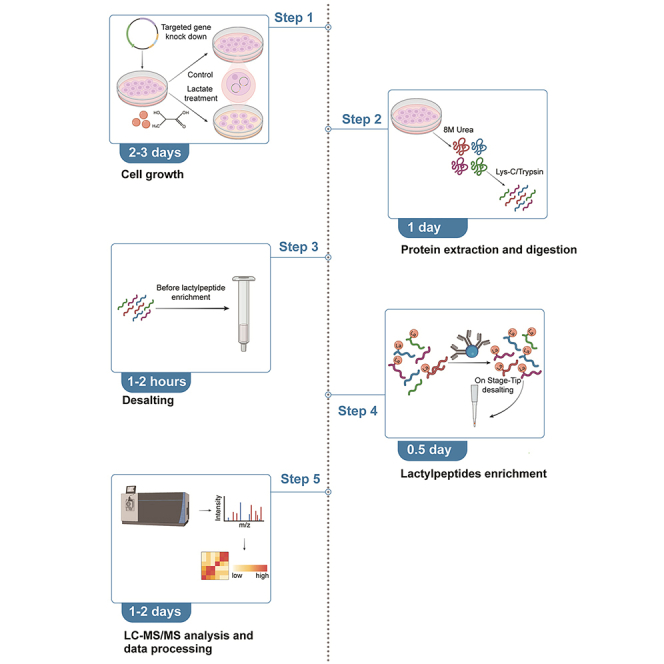

## Before you begin

### Preparation

This protocol describes quantitative analysis of protein lactylation in AARS1 and AARS2 (AARS1/2) knock down, AARS1/2 overexpression and wild type cells. For experimental uses, following materials should be prepared.

1. Synthesize mammalian cell expression vectors encoding AARS1/2 and siRNAs targeting AARS1/2.
2. Culture HEK293T cells (ATCC 3216) and HeLa cells (ATCC CCL-2) in DMEM (Fisher Scientific, Cat#31966047) supplemented with 10% (v/v) of fetal bovine serum (FBS Gibco, Cat#10270106), 100 mg/mL penicillin and streptomycin (Invitrogen, Cat#15140-122).
3. Maintain cell lines in a humidified incubator at 37°C, with 5% CO_2_. Regularly check for mycoplasma contamination using the EZ PCR Mycoplasma detection Kit (Biological Industries, Cat#20-700-20).
4. Refresh the cell medium at least every 48 h, by aspirating half of the old medium (taking care not to alter the spheroids) and adding an equal volume of fresh medium.
5. Calibrate LC-MS equipment and run quality control samples such as HeLa cell lysate standards to check if the LC-MS/MS performs as expected.

## Key resources table

**Table:** 

REAGENT or RESOURCE	SOURCE	IDENTIFIER
**Chemicals, peptides, and recombinant proteins**		

Dulbecco’s phosphate-buffered saline (PBS) (1 ×)	Thermo Fisher Scientific	Cat# 14040091
Dulbecco’s modified Eagle’s medium (DMEM)	Thermo Fisher Scientific	Cat# 31966047
Trypsin EDTA (0.05%)	Thermo Fisher Scientific	Cat# 25300054
Fetal bovine serum (FBS)	Gibco	Cat# 10270106
Penicillin-Streptomycin (10,000 U/mL)	Invitrogen	Cat# 15140-122
L-Lactic Acid	Aladdin	Cat# A2205202
Dimethyl sulfoxide (DMSO)	VWR	Cat# A3672
Ammonium bicarbonate	Cayman Chemical	Cat# 16894
Trypsin Gold, mass spectrometry grade	Promega	Cat# V5280
C18 Solid phase extraction disks	Empore	Cat# 2215-C18
33 μm Polymeric sorbent solid-phase extraction cartridges	Strata-X	Cat# 8B-S100-AAK
Anti-L-lactyllysine antibody-conjugated agarose beads	PTM Bio	Cat# PTM-1404
Tris (2-carboxyethyl) phosphinehydrochloride (TCEP)	Sigma-Aldrich	Cat# 24615-84-7
Iodoacetamide (IAA)	Sigma-Aldrich	Cat# 144-48-9
Formic acid (FA), LC-MS grade	Thermo Fisher Scientific	Cat# 28905
Lys-C, mass Spec grade	Promega	Cat# VA117A
Trifluoroacetic acid (TFA), eluent additive for LC-MS	Sigma-Aldrich	CAS# 76-05-1
Tris-HCl (tris(hydroxymethyl)aminomethane hydrochloride)	VWR	CAS# 1185-53-1
Urea, high purity	VWR	CAS# 57-13-6
Acetonitrile (ACN), Optima LC/MS grade	Thermo Fisher Scientific	CAS# A955-500
Methanol, Optima LC/MS grade	Thermo Fisher Scientific	CAS# 67-56-1
cOmplete, EDTA-free Protease Inhibitor Cocktail	Roche	CAS# 04693132001
Deacetylase Inhibitor Cocktail	MCE	CAS# HY-K0030

**Critical commercial assays**		

Polyethylenimine	Sigma-Aldrich	CAS# 9002-98-6
Pierce BCA Protein Assay Kit	Thermo Fisher Scientific	Cat# 23225
Lipofectamine RNAiMAX Transfection 1070 Reagent	Thermo Fisher Scientific	Cat# 13778150
EZ PCR Mycoplasma detection Kit	Biological Industries	Cat# 20-700-20

**Experimental models: Cell lines**		

Human cell line, HEK293T	ATCC	Cat# CRL-3216
Human cell line, HeLa	ATCC	Cat# CRM-CCL-2

**Oligonucleotides**		

Human AARS1 siRNA #1 5′–3′-sequence GCAGTGAGATCCACTACGA	Ribo Life Science	N/A
Human AARS1 siRNA #2 5′–3′-sequence GTTTGGCATTCCCATTGAA	Ribo Life Science	N/A
Human AARS2 siRNA #1 5′–3′-sequence GGAACCTGGTCTTCATGCA	Ribo Life Science	N/A
Human AARS2 siRNA #2 5′–3′-sequence GTTTGGCATTCCCATTGAA	Ribo Life Science	N/A
Negative control siRNA	Ribo Life Science	N/A

**Recombinant DNA**		

pLV-AARS1-Myc	N/A	N/A
pLV-AARS2-Myc	N/A	N/A

**Software and algorithms**		

MaxQuant	Tyanova et al.^2^	MaxQuant v2.0.3.0
Microsoft Excel	Microsoft Corporation	N/A
UniProt database human	N/A	https://www.uniprot.org/proteomes/UP000005640

**Other**		

CO_2_ incubator	Thermo Fisher Scientific	Cat#51033770
SpeedVac vacuum concentrators	Thermo Fisher Scientific	Cat#SPD111V-115
Benchtop freeze dryers	Labconco	Cat# 10-400-021
Microcentrifuge	BMG LABTECH	Cat# 22620667
Nunc EasYDish dishes	Thermo Fisher Scientific	Cat# 150464
Axygen 1.5 mL MaxyClear Snaplock	Axygen	Cat# MCT-150-C
Axygen 1000 μL pipet tips	Axygen	Cat# T-1000-B
Axygen 10 μL microvolume pipet tips	Axygen	Cat# T-300-R-S
Axygen 200 μL Universal Fit pipet tip	Axygen	Cat# T-200-C
Thermo Scientific Exploris 480 mass spectrometer	Thermo Fisher Scientific	Cat# BRE725533
Thermo Scientific EASY-nLC 1200 instrument	Thermo Fisher Scientific	Cat# LC140

## Materials and equipment

**Table:** Cell Culture Medium

Reagent	Final concentration	Amount
DMEM	N/A	500 mL
Fetal Bovine Serum	10%	50 mL
Penicillin/ streptomycin (10,000 U/ mL)	100 U/ mL	5 mL
Total	N/A	555 mL

***Note:*** Cell Culture Medium can be stored at 4°C for 1 month.

**Table:** Denaturing Lysis Buffer

Reagent	Final concentration	Amount
Urea	8 M	240 mg
Tris/HCl pH 8.5 (1 M)	100 mM	50 μL
LC-MS grade H2O	N/A	270 μL
Protease Inhibitor (100×)	N/A	5 μL
Deacetylase Inhibitor (100×)	N/A	5 μL
Total	N/A	500 μL

***Note:*** Urea is unstable. Prepare freshly before each use.

**Table:** Lactylpeptide Binding buffer

Reagent	Final concentration	Amount
Tris/HCl pH 8.0 (1 M)	50 mM	2.5 mL
NaCl (5 M)	100 mM	1 mL
EDTA (500 mM)	1 mM	100 μL
NP-40 (20%)	0.5% (v/v)	1.25 mL
LC-MS grade H_2_O	N/A	45.15 mL
Total	N/A	56 mL

***Note:*** Lactylpeptide Binding buffer can be stored at room temperature for 1 month.

**Table:** Lactylpeptide Wash buffer

Reagent	Final concentration	Amount
Tris/HCl pH 8.5 (1 M)	50 mM	2.5 mL
NaCl (5 M)	100 mM	1 mL
LC-MS grade H_2_O	N/A	46.5 mL
Total	N/A	50 mL

***Note:*** Lactylpeptide Wash buffer can be stored at room temperature for 1 month.

**Table:** Lactylpeptide Elution buffer

Reagent	Final concentration	Amount
FA	5% (v/v)	500 μL
LC-MS grade H_2_O	N/A	10 mL
Total	N/A	10 mL

***Note:*** As the concentration of FA is relatively high, Lactylpeptide Elution buffer can be stored at room temperature for 1 week.

**Table:** Ammonium Bicarbonate Buffer

Reagent	Final concentration	Amount
Ammonium Bicarbonate	400 mM	1.56 g
LC-MS grade H_2_O	N/A	50 mL
Total	N/A	50 mL

***Note:*** 400 mM ammonium bicarbonate (ABC) buffer stock can be stored at room temperature for 1 month. Dilute ABC buffer to 50 mM before use.

**Table:** Desalting activation buffer

Reagent	Final concentration	Amount
LC-MS grade Methanol	100% (v/v)	10 mL
Total	N/A	10 mL

***Note:*** Methanol is highly flammable and harmful if swallowed. Keep away from heat, hot surfaces, sparks, open flames and other ignition sources.

***Note:*** Desalting activation buffer can be stored at room temperature for 1 month.

**Table:** SPE column equilibration buffer

Reagent	Final concentration	Amount
40% TFA	0.1% (v/v)	25 μL
LC-MS grade H_2_O	N/A	9.975 mL
Total	N/A	10 mL

***Note:*** TFA is highly corrosive and volatile. Handle in a fume hood and wear gloves and goggles when operate experiments.

***Note:*** SPE column equilibration buffer can be stored at room temperature for 1 month.

**Table:** SPE elution buffer

Reagent	Final concentration	Amount
ACN	80% (v/v)	8 mL
40% TFA	0.1% (v/v)	25 μL
LC-MS grade H_2_O	19.9% (v/v)	1.975 mL
Total	N/A	560 mL

***Note:*** TFA is highly corrosive and volatile. ACN is highly flammable. Handle in a fume hood and wear gloves and goggles when operate experiments. Check pH before use and ensure pH is below 1.

***Note:*** SPE elution buffer can be stored at room temperature for 1 month.

**Table:** Stage tip desalting buffer A

Reagent	Final concentration	Amount
FA	0.1% (v/v)	10 μL
LC-MS grade H_2_O	N/A	9.99 mL
Total	N/A	10 mL

***Note:*** Stage tip desalting buffer A can be stored at room temperature for 1 month.

**Table:** Stage tip desalting buffer B

Reagent	Final concentration	Amount
FA	0.1% (v/v)	10 μL
ACN	99.9% (v/v)	9.99 mL
Total	N/A	10 mL

***Note:*** Stage tip desalting buffer B can be stored at room temperature for 1 month.

**Table:** Stage tip desalting buffer C

Reagent	Final concentration	Amount
ACN	40% (v/v)	4 mL
FA	0.1% (v/v)	10 μL
LC-MS grade H_2_O	59.9% (v/v)	5.99 mL
Total	N/A	10 mL

***Note:*** Stage tip desalting buffer C can be stored at room temperature for 1 month.

**Table:** LC mobile phase A

Reagent	Final concentration	Amount
ACN	2% (v/v)	500 μL
FA	0.1% (v/v)	25 μL
LC-MS grade H_2_O	97.9% (v/v)	24.975 mL
Total	N/A	25 mL

**Table:** LC mobile phase B

Reagent	Final concentration	Amount
ACN	80% (v/v)	20 mL
FA	0.1% (v/v)	25 μL
LC-MS grade H_2_O	20% (v/v)	4.975 mL
Total	N/A	25 mL

***Note:*** Degassing of LC mobile phase for 30 min using ultrasound.

***Note:*** LC mobile phase can be stored at room temperature for 1 week.

**CRITICAL:** The concentration of ACN should not greater than 95%, otherwise it will damage the HPLC instrument.

**Table:** Reduction buffer (TCEP buffer)

Reagent	Final concentration	Amount
TCEP	1 M	2.50187 g
LC-MS grade H_2_O	N/A	10 mL
Total	N/A	10 mL

**Table:** Alkylation buffer (IAA buffer)

Reagent	Final concentration	Amount
IAA	500 mM	924.82 mg
LC-MS grade H_2_O	N/A	10 mL
Total	N/A	10 mL

***Note:*** Reduction and Alkylation buffer can be stored at −20°C for 1 month.

**Table:** L-lactic acid stock

Reagent	Final concentration	Amount
L-lactic acid	10 M	9.008 g
MilliQ water	N/A	10 mL
Total	N/A	10 mL

***Note:*** L-lactic acid stock can be stored at −20°C for 1 month.

## Step-by-step method details

### Cell culture

**Timing: 2 days**

This part describes the preparation of a HEK293T cell culture for transfection.

1. Pre-warm DMEM, PBS and trypsin-EDTA in a 37°C water bath.
2. Cultivate HEK293T cells in a 10 cm dish under humidified conditions with 5% CO_2_ at 37°C using DMEM medium until they reach 80%–90% confluency.
3. Aspirate the growth medium and gently wash the cells twice with 2 mL of sterile PBS.
4. Aspirate the PBS and detach the cells by adding 1 mL of trypsin-EDTA. Incubate at 37°C until the cells have detached (approximately 30 s).
5. To neutralize trypsin-EDTA, add 2 mL of growth medium and resuspend by pipetting until all the cells have been stripped off from the bottom of the dish.
6. Transfer the cell suspension to a 15 mL Falcon tube and pellet the cells by centrifugation for 3 min at 200 *× g* and between 22°C–25°C.
7. Aspirate the supernatant and resuspend the cell pellet in 1 mL of fresh growth medium.
8. Seed one-quarter of the cell suspension into a new 10 cm dish and add an additional 8 *mL* of fresh growth medium.
9. Culture the cells until they achieve optimal confluency for transfection.

***Optional:*** To investigate the impact of a specific gene knockdown on lactylation, HEK293T can be transfected with siRNA after seeding. In this protocol, we designed siRNAs to knock down the expression of AARS1 and AARS2. The sequences of indicated siRNAs are provided in key resources table - Oligonucleotides.

### Cell transfection

**Timing: 60–120 min**

AARS1/2 siRNA, AARS1/2 constructs or control plasmids are transfected into HEK293T cells either separately or in combination. Each treatment should be prepared with at least two 10 cm dish of cells to supply sufficient protein for lactylpeptides enrichment (refer to part 7). In this protocol, we use polyethylenimine (PEI) for the transfection of AARS1/2 constructs or control plasmids and Lipofectamine RNAiMAX for siRNA transfection. The required amounts of siRNA, DNA constructs and PEI for transfection are specified in Table 1.

10. Culture the cells until they reach 40%–50% confluency prior to transfection.
11. Dilute the appropriate amount of siRNA or plasmids as indicated in Table 1 using DMEM medium without serum.

***Note:*** siRNA should be resuspended in RNase-free water and stock in 20–100 μM.

12. Dilute the appropriate amount of Lipofectamine or PEI as indicated in Table 2 using DMEM medium without serum. Combine it with diluted siRNA or plasmids and incubate for 20 min at room temperature.
13. Add the DNA mix dropwise to the corresponding plates.
14. Incubate the transfected cells in the incubator (37°C, 5% CO_2_) for 6 h.
15. Aspirate the medium and replace it with fresh DMEM medium.

***Note:*** It is essential to include controls in all siRNA experiments to assess the efficiency of targeted gene knockdown.

**Table 1:** Example amounts of transfected AARS1/2 siRNA, AARS1/2 constructs and control plasmids

Culture format	Total volume/mL	DMEM without serum (mix with siRNA/plasmid)/μL	DMEM without serum (mix with PEI)/μL	siRNA/μL	Final siRNA concentration/nM	Plasmids/μg	PEI or Lipofectamine RNAiMAX Reagent /μL
100 mm plates	8	400	400	20	50	10	20
60 mm plates	4	200	200	10	50	5	10
6-well	2	100	100	5	50	2	4
12-well	1	50	50	2.5	50	1	2

**Table 2:** The setup of LC gradient

Time (mm:ss)	Duration (mm:ss)	Flow (nL/min)	Mixture (%B)
00:00	00:00	450	3
02:00	02:00	450	6
66:00	64:00	450	27
82:00	16:00	450	35

### Lactate stimulation

**Timing: 1 day**

To conduct a more comprehensive comparison of the variation of protein lactylation among different experiments, we established a parallel group in addition to the normally cultured cells, in which lactate is added to the cell culture medium to stimulate the level of lactylation.

16. After replacing with fresh DMEM medium, add the lactate solution to the medium at a final concentration of 25 mM.
17. Incubate the cells in the incubator for 24–36 h.

### Cell harvesting and lysis

**Timing: 2 h**

18. Carefully aspirate the medium from the cells.
19. Rinse the cells twice by adding 2 mL of ice-cold PBS each time.
20. Detach the cells by adding 1 mL of PBS and pipetting until all cells are dislodged from the bottom of the dish. Collect the cells into a new 1.5 mL Eppendorf tube.

***Note:*** Use cell scraper to detach the cells if cells are too adherent to be dislodged from the bottom of the dish by pipetting.

21. Pellet the cells by centrifugation at 900 *× g* for 10 min at 4°C.
22. Aspirate the supernatant.

***Note:*** If you plan to perform SILAC experiments, count cells and mix equal amounts of heavy isotope-labeled cells and control cells in this step.

23. Resuspend the cell pellet with 500 μL denaturing lysis buffer.
24. Reduce cell lysate viscosity by sonicating using a contact ultrasonic horn (2 min total; pulse pattern: ON for 2 s, OFF for 2 s; amplitude set at 35%).

**CRITICAL:** The size of ultrasonic horn and duration of ultrasonication should be selected on the basis of amount of protein and the volume of protein solution.

**CRITICAL:** An ice bath should be applied to prevent protein degradation during sonicating.

25. Centrifuge samples at 13000 *× g* for 15 min at 4°C to remove insoluble materials. Collect supernatant into a new 1.5 mL Eppendorf tube.

### Protein digesting

**Timing: 0.5 day**

26. Determine the protein concentration of cell lysate using the BCA assay and separate 2 mg of proteins for digestion.

***Note:*** The standard curve for the BCA assay should cover the entire range of expected concentrations of samples. The standard curve needs to be established for each sample determination.

27. Precipitate protein.
  a. Dilute the lysate solution to 4 M urea with 100 mM Tris-HCl, pH 8.5.
    **CRITICAL:** At the concentration of 8 M, urea will precipitate out after adding acetone and prevent the protein digestion efficiency.
  b. Add ice-cold acetone to the lysate solution and incubate the mixture at −20°C for 30 min, using a volume of acetone that is six times of the lysate solution.
  c. Pellet the precipitated proteins by centrifugation at 13000 × *g* for 30 min at 4°C.
    **Pause point:** Samples can be stored at this point for up to 1 month at −80°C.
28. Digest with Lys-C.
  a. Resuspend the protein pellet with 500 μL of 50 mM ABC buffer.
    ***Note:*** The pH of 50 mM ABC buffer should be 8.0. Prepare new buffer if the pH deviates too much.
  b. Add Lys-C to the lysate at a final concentration of 10 ng/μL.
  c. Incubate at 37°C for 3–4 h.
    **CRITICAL:** Keep protein pellet suspended in an incubator shaker set at 1500 rpm.
29. Digest with trypsin.
  a. Add Trypsin into the reaction mixture at an enzyme-to-substrate ratio of 1:100.
  b. Incubate at 37°C for 12 h.
30. Peptide reduction and alkylation
  a. Add 5 μL 1 M TCEP buffer to the protein digestion solution. Mix thoroughly and incubate at 25°C for 15 min.
  b. Add 10 μL of 500 mM IAA buffer to the protein digestion solution. Mix and incubate at 25°C for 20 min in darkness.

**CRITICAL:** IAA is photo-labile and alkylation should be conducted in darkness.

31. Terminate digestion by adding TFA to a final concentration of 1%.
32. Centrifuge samples at 13000 *× g* for 5 min at room temperature to remove insoluble materials.
33. Transfer the supernatant into a new 1.5 mL Eppendorf tube for peptide desalting.

### Peptide desalting

**Timing: 0.5 day**

We use reverse-phase Strata-X 33 μm Polymeric sorbent solid-phase extraction cartridges from Phenomenex. The size of the cartridge should be chosen on the basis of the amount of digested protein, corresponding to approximately 10% (wt/wt) of the weight of the packing material. In this protocol, we recommend using a 10 mg Strata-X cartridge (10 mg polymeric sorbent with a volume of 1 mL). Gravity flow is adequate for allowing samples to pass through the cartridge if the insoluble materials are removed thoroughly by centrifugation. A vacuum manifold or air pressure can be employed to enhance solvent flow rates and provide more uniform peptide loading and elution.

34. Condition the cartridge with 1 mL desalting activation buffer.

**CRITICAL:** Do not drain the cartridges before elution step. Remain a laminate of fluid above cartridges in the end of each step.

35. Equilibrate the cartridge with 2 mL SPE equilibration buffer.
36. Load the collected peptide solution.

***Note:*** The cartridges may turn yellow after sample passage, which indicates the reduction and alkylation had been conducted.

37. Wash with 2 mL SPE equilibration buffer.
38. Elute with 1 mL SPE elution buffer and collect eluate in a new 1.5 mL Eppendorf tube.
39. Separate 50 μL of the eluate (100 μg peptides of the cell lysate) and dry it with a SpeedVac centrifuge.

***Note:*** The separated peptides will be used for proteome analysis.

40. Freeze the eluate with liquid nitrogen and subsequently lyophilize it.

**CRITICAL:** Lyophilize should be thoroughly and remove all residual acid from sample for the next lactylpeptide enrichment step.

***Note:*** The lyophilized powder should be yellowish white and fluffy. If lyophilize is unavailable, dry the eluate using a SpeedVac centrifuge.

**Pause point:** Samples can be stored at this point for up to 1 month at −80°C.

### Lactylpeptide enrichment

**Timing: 0.25 day**

**Figure 1:**
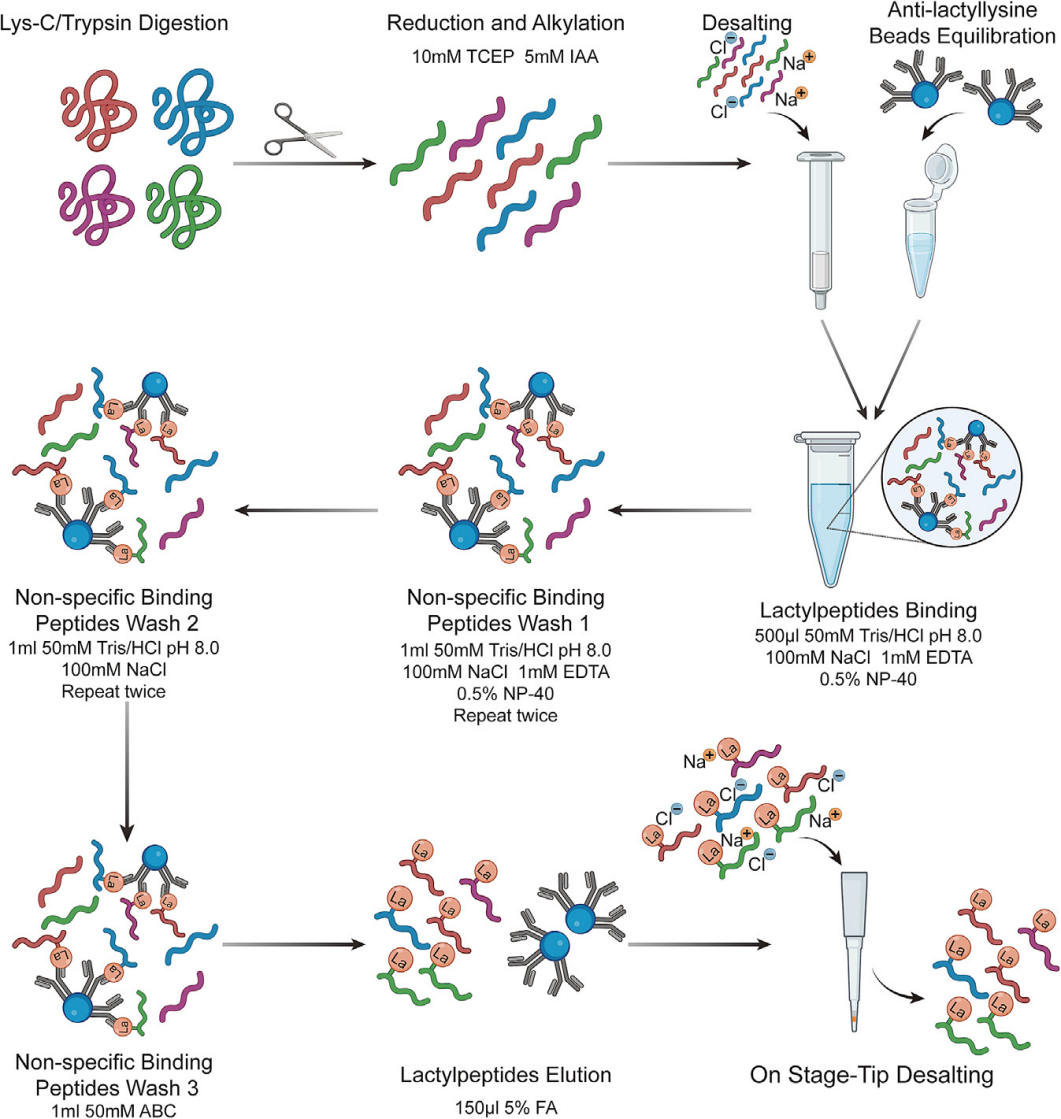
Schematic representation of lactylpeptide enrichment utilizing anti-lactyllysine antibody-conjugated beads

We utilize PTM-Bio Anti-L-Lactyllysine Antibody Conjugated Agarose Beads (PTM-1404). The amounts of beads should be determined based on the amounts of digested proteins. In this protocol, for every 2 mg of protein digest, it is recommended to use 10 μL of anti-lactyllysine beads Figure 1. During each step, centrifuge in microcentrifuge at 1000 *× g* for 1 min at 4°C to pellet the beads.

***Note:*** Before formal experiments, researchers should apply standards such as HeLa cell lysate to perform a pre-experiment and make sure the enrichment efficiency of Anti-L-Lactyllysine Antibody Conjugated Agarose Beads is consistent between batches and vendors.

41. Resuspend the lyophilized peptides in 1 mL lactylpeptide binding buffer. Vortex or sonication is necessary to ensure complete dissolution of the peptides.

***Note:*** Check the pH of the solution before peptide enrichment. pH should be approximately 8.0.

42. Clarify solution by centrifugation at 13000 *× g* for 10 min at 4°C.
43. Aspirate 10 μL anti-lactyllysine beads and wash the beads with 1 mL Lactylpeptide binding buffer. Pellet the beads through centrifugation.
44. Transfer clarified peptide solution into a tube containing anti-lactyllysine beads and incubate for 4 h while rotating at 4°C.

***Note:*** The incubation time should be no less than 4 h to ensure the lactylpeptides enrichment efficiency. We also don’t recommend incubation overnight which will lead to more non-specific binding.

45. Pellet the beads by centrifugation.

***Note:*** Collect supernatant as a backup in case lactylpeptide enrichment failed. The supernatant can be stored for up to 1 month at −80°C.

46. Wash the beads twice with 1 mL lactylpeptide binding buffer for 10 min while rotating at 4°C.
47. Wash beads twice with 1 mL lactylpeptide wash buffer for 10 min while rotating at 4°C.
48. Perform a final wash with 1 mL 50 mM ammonium bicarbonate buffer for 10 min while rotating at 4°C.
49. Elute lactylpeptide from the beads.
  a. Resuspend the beads with 100 μL of 1% TFA, allowing them to stand at room temperature for 10 min. Pellet the beads by centrifugation and collect supernatant into a new 1.5 mL Eppendorf tube.
  b. Resuspend the beads with 50 μL of 1% TFA and incubate 10 min with rotator. Pellet the beads by centrifugation and combine supernatant with the first elution.

***Note:*** Solution can be filtered with a 0.22 μm filter to remove trace beads.

### Stage-tip desalting

**Timing: 2 h**

The eluate of lactylpeptides needs to be cleaned up for mass spectrometry analysis using a C18 stage-tip. In this protocol, we employ Empore C18 Solid Phase Extraction Disks (2215-C18) to make up stage-tips. Three layers of disks are piled in a pipette tip and compacted with another pipette tip. During each step, centrifuge in a microcentrifuge at 1200*× g* to facilitate the passage of the solution through the column.

***Note:*** The centrifugation speed should be adjusted according to your stage-tip and make sure the solvent flow is maintained at 5 μL/min.

50. Condition a stage tip with 20 μL desalting activation buffer.
51. Wash the stage tip with 20 μL Stage tip desalting buffer B.
52. Equilibrate the stage tip with 20 μL Stage tip desalting buffer A
53. Force the lactylpeptide eluate through the stage-tip.
54. Desalt the sample with 20 μL Stage tip desalting buffer A.
55. Transfer the stage tip to a fresh Eppendorf tube.
56. Elute peptides with 20 μL Stage tip desalting buffer C. Collect the flowthrough.
57. Elute peptides with 20 μL Stage tip desalting buffer B. Combine the flowthrough with that from the first elution.
58. Dry the eluate in a SpeedVac centrifuge.

**Pause point:** Samples can be stored at this point for up to 1 week at −80°C.

### LC-MS analysis

**Timing: weeks**

In this part, we describe the setup of an Orbitrap Exploris 480 coupled with Easy-nLC 1200 system. The HPLC and Mass spectrometry method parameters described below may differ from other instruments.

59. Prepare LC-MS/MS equipment.
  a. Install fresh mobile solvent A and solvent B in sufficient amounts. Sonication of both solvent A and B is required to clean the bubble.
  b. Flush air of pump A, pump B and pump S with mobile phase buffer. The flush threshold is 10 μL.
  c. Activate the analytical column with 90% solvent B buffers for 10 μL.
  d. Equilibrate the analytical column by running a blank sample. Ensure the mobile phase flow path is smooth.

***Note:*** For installing mobile solvent, flushing air and activating analytical column, it may take 1 h. For running a blank sample, it may take 30 min.

60. Prepare Klac enrichment samples.
  a. Dissolve enriched Klac peptides in 5 μL of 0.1% formic acid (solvent A).
  b. Centrifuge the peptides at 12, 000*× g* for 20 min at 4°C to remove insoluble substance.
  c. Transfer 4.5 μL of the supernatant into autosampler vials.
  d. Load 4 μL samples into an Easy-nLC 1200 HPLC.
61. Prepare proteome samples.
  a. Dissolve separated peptides in step 39 with 20 μL of 0.1% formic acid (solvent A).
  b. Centrifuge the peptides at 12, 000*× g* for 20 min at 4°C to remove insoluble substance.
  c. Transfer 18 μL of the supernatant into autosampler vials.
  d. Load 1 μL samples into an Easy-nLC 1200 HPLC.
62. LC gradient settings are provided in Table 2.
63. MS parameters
  Setup the mass spectrometer method for the Orbitrap Exploris 480 with the following parameters.
  a. Positive ion mode.
  b. Spray voltage at 2.0 kV.
    ***Note:*** If the steel emitter is stained or worn and the spray is not stable, increase spray voltage to 2.1 kV.
  c. Heated capillary temperature at 320°C.
  d. Set full MS resolution at 60,000 with a maximum injection time of 25 ms.
  e. Set the MS1 mass range at 350–1400 with an AGC target at 1e6.
  f. Set the HCD fragment spectra resolution at 15,000 with a maximum injection time of 22 ms, using the Top20 method.
  g. Set MS2 mass range at 200–1400 m/z with an AGC target at 5e4.
  h. Set the maximum injection time at 45 ms.
  i. Set the isolation window at 1.6 m/z and the normalized collision energy at 27%.
    ***Note:*** The best normalized collision energy may differ from other instruments.

### Data processing

**Timing: 0.5 day**

**Figure 2:**
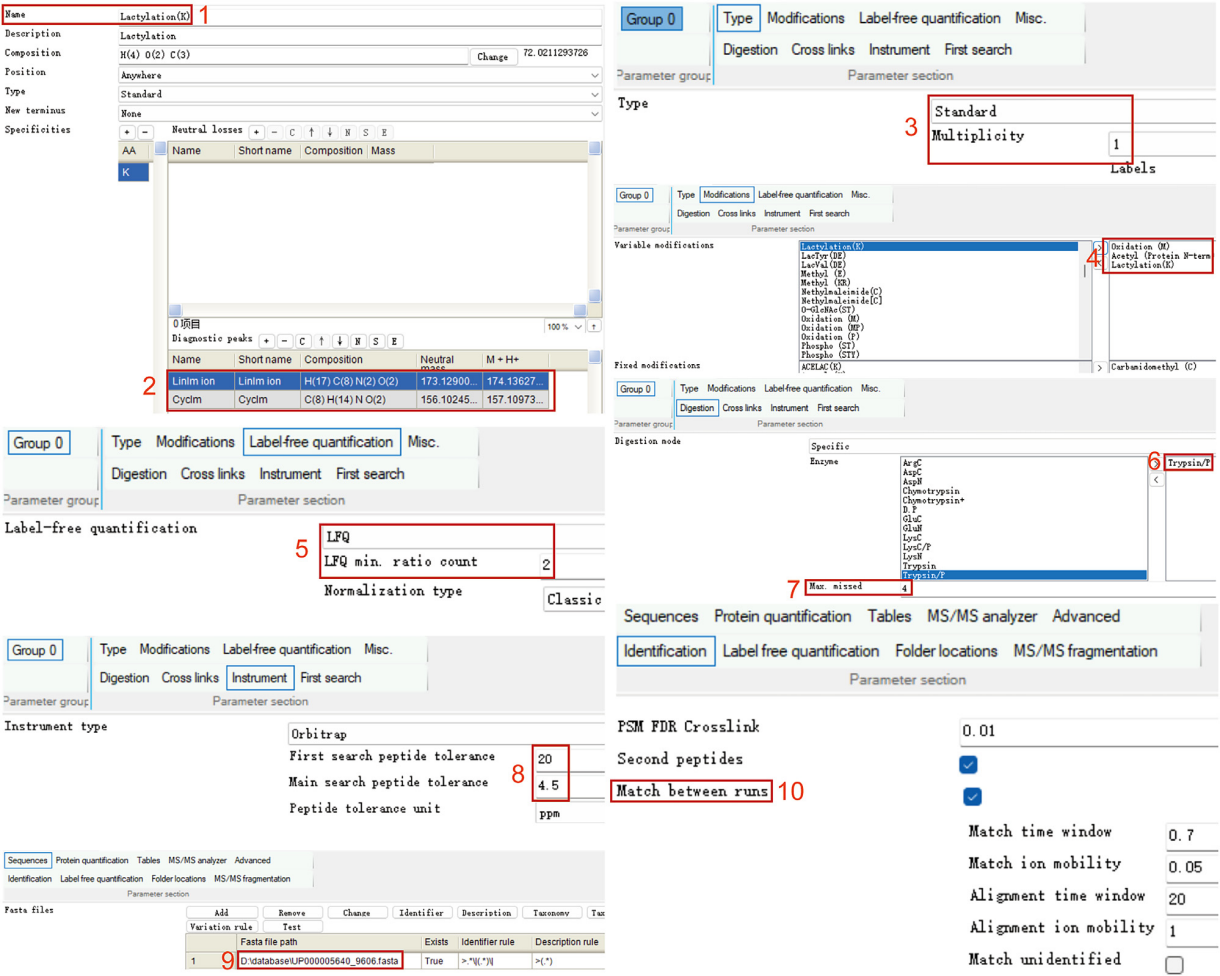
Configuration of MaxQuant parameters for the identification and quantification of lactylpeptides

Here we outline the processing of raw files using MaxQuant. We provide detailed steps for setting up a new modification and the parameters for label-free quantification. In this protocol, we use the MaxQuant (version 2.0.3.0), with all acquired raw files being searched against the UniProtKB human complete proteome sequence database. Please refer to the accompanying Figure 2 for a workflow that details the parameters used in MaxQuant for analyzing these data. Other softwares such as Proteome Discoverer, pQuant and MSFragger may also be used for MS raw files processing and label-free quantification.

64. Launch the MaxQuant software.
65. Click on “Configuration” and select “Add” to create a new modification for lactylation. Enter “Lactylation (K)” in the “Name” field and “LysineLactylation” in the description field. In “Composition”, choose “H(4) O(2) C(3)” and “Change” will automatically show “72.0211293726”. For "Position," select “Not C-term,” set "Type" to “Standard,” choose “None” for "New terminus," and designate "Specificities" as “Lysine.”

***Note:*** Lactylation on lysine prevents trypsin digestion of lysine C-terminus. Therefore, we suggest to select “Not C-term” for Lactylation (K).

***Note:*** In reference of Wan et al., [1]^3^ we recommend configuring two diagnostic peaks. Set “Linlm ion” consisting of “H(17) C(8) N(2) O(2)” and “Cyclm ion” consisting of “H(14) C(8) N(1) O(2)”.

66. Restart MaxQuant to load the new set modification.
67. Select Raw data, then click on “Load” to load all raw data files.
68. Select each row of raw data, then click “Set Experiment” to assign name for each raw data.
69. Click on “Group-specific parameters” and select “Standard” and the “Multiplicity” is 1 for label-free quantification.
70. Click on “Modification” and select carbamidomethylation of cysteines as fixed modification, oxidation of methionine, acetylation of any protein N-terminal and lactylation of lysine as variable modifications.
71. We use LFQ with a minimum ratio count of 2 when performing label-free quantification.
72. Select the digestion mode with “specific” and choose “Trypsin/P” for the enzyme. The maximum number of missed cleavages should be set to 4.
73. First search peptide tolerance is 10 ppm and the main search peptide tolerance is 4.5 ppm.
74. In the Global parameters tab, click on “sequence” and upload a FASTA file of the Homo proteome downloaded from Uniport (refer to key resources table - Software and algorithms).
75. Click on “Identification” and choose “Match between runs”.
76. Select suitable “Number of processors” based on the threads of CPUs.

***Note:*** All result files will be located in the folder “…\combined\txt” as tab-delimited text files. Data processing and annotation are performed by manual operation.

***Note:*** Raw data belonging to Klac enrichment samples and proteome samples should be analyzed separately. For proteome samples, remove lactylation of lysine from variable modifications (step 70) and remain other parameters of MaxQuant setup.

77. Open “Lactylation(K)” and “proteinGroups” with Microsoft Excel and remove the reverse and potential contaminant hits.

***Note:*** Align the lactylation sites to the identified proteins in “proteinGroups” file and use the differences of protein expression calculated by proteome analysis to normalize the differences of lactylation levels calculated by Klac peptide analysis.

## Expected outcomes

**Figure 3:**
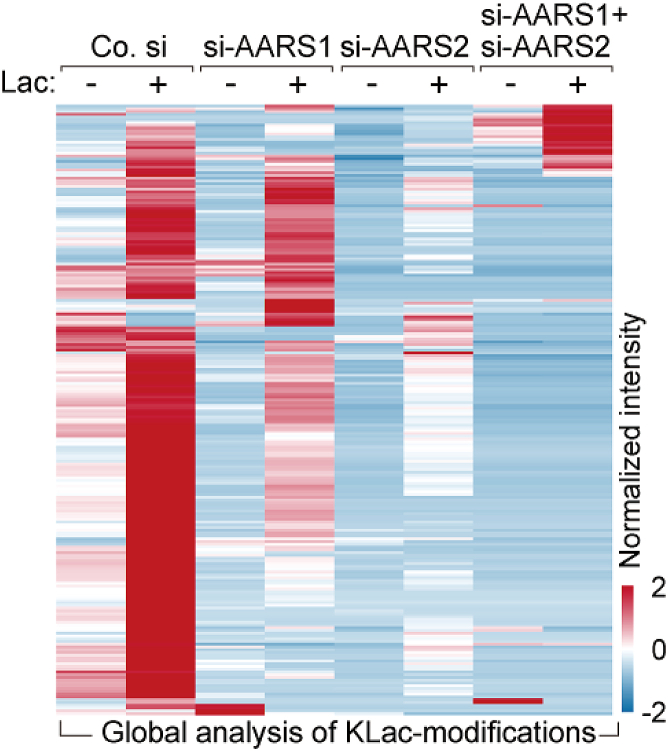
Heatmap showing color-coded intensity levels of global lysine lactylome in control, AARS1 and/or AARS2 depleted (si-AARS1/si-AARS2) HEK293T cells treated with PBS (Lac “-“) or L-lactate (Lac “+”) for 24 h

**Figure 4:**
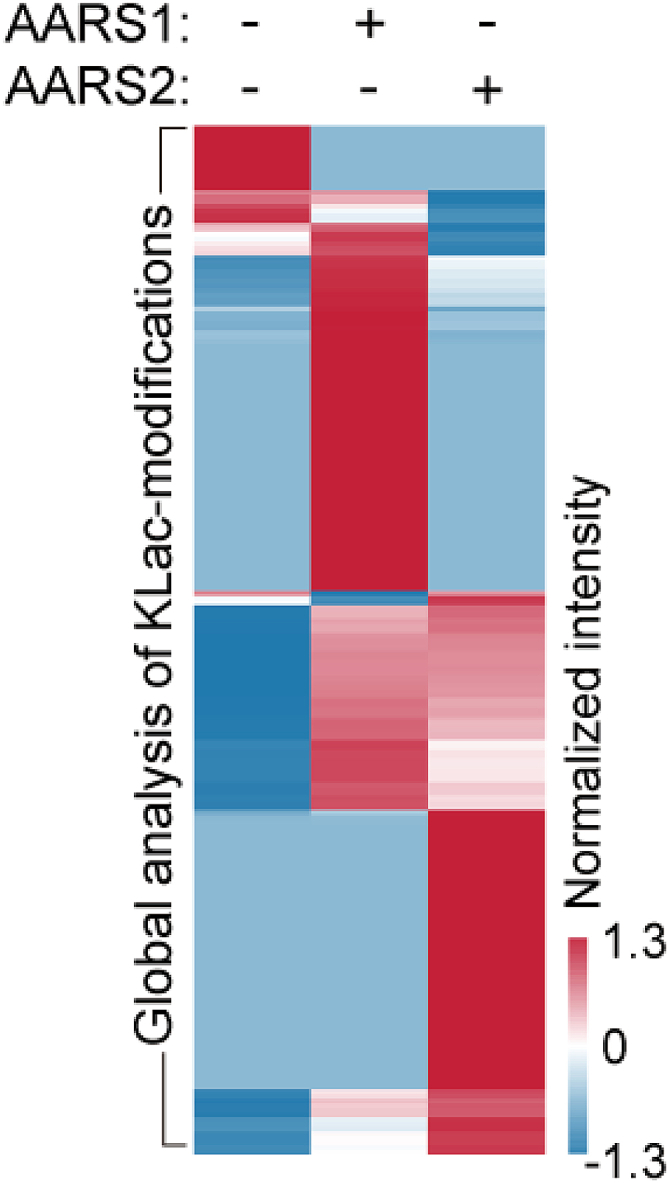
Heatmap showing color-coded intensity levels of global lysine lactylation in HeLa cells transfected with control (AARS1 -/AARS2 -) or AARS1/AARS2 expressing plasmid (AARS1 +/AARS2 +) as indicated

In this protocol, we performed a workflow to efficiently enrich lactyllysine peptides. Based on Mass spectrometry, we can comprehensively profile lactyllysine modification and quantify the modification levels across control cells, AARS1/2 knockdown cells and AARS1/2 overexpressing cells. The Table 3 presents the amounts of identified lactyllysine modification sites in indicated cells using the workflow described in this protocol. Additionally, the Figures 3 and 4 illustrates the heatmap depicting differences in lactyllysine modifications in each group.

**Table 3:** The amounts of identified PSMs, peptides, lactyllysine modification sites and lactyllysine modification proteins in indicated cell lines

Cell lines	Number of lactylpeptide PSMs	Number of lactylpeptide	Number of lactylsites	Number of Lactylproeins
HEK 293T	67550	45773	1704	798
HeLa	27979	22345	405	272

## Limitations

In this protocol, we describe a workflow for the efficient enrichment of lactylpeptides and profiling of lactylation using LC-MS and anti-L-lactyllysine antibody conjugated agarose beads. L-lactate is a metabolite generated by glycolytic pathway. However, in certain metabolic disorders, such as diabetes and neurodegenerative diseases, D-lactate, a geometric isomer of L-lactate, will gain of generation due to the accumulation of glyoxalase.^4^^,^^5^^,^^6^^,^^7^ To explore the function of lactate and lactylation in cellular process further, methods which specific target D-lactate and D-lactylation are needed to incorporate with the method of this protocol.

Current research on the regulation of lactylation remains limited, and broad spectrum delactylase inhibitor is not available. In this protocol, we employ urea as a denaturing agent to deactivate potential delactylases during cell lysis while simultaneously adding deacetylase inhibitors to minimize loss of lactylation. With the further exploration of lactylation regulation, it will be imperative to develop more specific delactylase inhibitors.

## Troubleshooting

### Problem 1

Protein concentration is low after cell lysis.

### Potential solution

Assess the viscosity of the protein solution after sonication. If it appears viscous, consider extending the duration or increasing the power of the sonication. Meanwhile, you can treat the samples with Benzonase to mitigate the influence of DNA content.

### Problem 2

Peptide recovery is low after desalting.

### Potential solution

Ensure that the amount of peptides does not exceed the maximum capacity of the cartridges. Verify that the pH of the desalting buffer falls within a range of 1 to 1.5. If the pH is out of this range, discard the expired buffers and prepare fresh buffers. Confirm that cartridges do not dry out prior to peptide elution.

### Problem 3

The rate of sample flow through cartridges is slow or even blocked off when desalting.

### Potential solution

Check the protein concentration and make sure the amount of loaded peptides does not exceed the maximum of the capacity of cartridges. Before desalting, centrifuge the digestion solution to pellet the insoluble materials and aspirate the supernatant carefully to avoid carrying out any precipitate.

### Problem 4

Recovery of lactylpeptides is low.

### Potential solution

Given that lactate is toxic to cells, monitor cellular health regularly during incubation with lactate, ensuring cells maintain normal morphology before harvesting. The lactylpeptide elution buffer should be prepared fresh in case peptides cannot be completely stripped from beads.

### Problem 5

Chromatogram peaks are disconnected or evenly distributed.

### Potential solution

Check whether the emitter is stained and substitute with a new one if necessary. Prepare fresh solvent A and B to prevent the delay of chromatogram peaks caused by volatilization of ACN.

## Resource availability

### Lead contact

Further information and requests for resources and reagents should be directed to and will be fulfilled by the lead contact, Prof. Dr. Long Zhang (l_zhang@zju.edu.cn).

### Technical contact

Questions about the technical specifics of performing the protocol should be directed to and will be answered by the technical contact, Yu Ran (yuran@zju.edu.cn).

### Materials availability

This study did not generate new unique reagents.

### Data and code availability

The published article, Li et al.,^1^ includes all datasets analyzed during this study.

## Acknowledgments

The current work was supported by Chinese National Natural Science Funds (31925013, 32125016, 52425305, U20A20393, W2411011, T2321005, 31701234, 91753139, 92169122, 82473119, 31671457, 31870902, 32070907, 32100699, and 31871405); a program from the Ministry of Science and Technology of China (2021YFA1101000, 2022YFA1105200, 2023YFA1800200, and 2024YFC2707400); a Key R&D Program of Zhejiang Province (2024C03142); and the Joint Project of Pinnacle Disciplinary Group, the Second Affiliated Hospital of Chongqing Medical. We acknowledge Jiansheng Guo, Wei Yin, and Shuang shuang Liu from the core facility platform of Zhejiang University School of Medicine for technical support.

## Author contributions

L. Zhang and Y.R. participated in the initial conceptualization. Y.R., Q.Y., and L. Zhou designed and carried out the experiments. Y.R., H.L., and Q.Y. analyzed the data. Y.R., L. Zhou, H.L., and Q.Y. wrote the first draft of the manuscript. L. Zhang and B.Y. reviewed and edited the manuscript. B.Y. and L. Zhang supervised the entire study.

## Declaration of interests

The authors declare no competing interests.
